# Two-year evaluation of a nano-hybrid and a bulk-fill resin composite: a randomized, double-blind split-mouth clinical study

**DOI:** 10.1007/s00784-024-05592-8

**Published:** 2024-03-11

**Authors:** Funda Çağırır Dindaroğlu, Ece Yılmaz

**Affiliations:** 1 İzmir Katip Çelebi University, School of Dentistry, Department of Pediatric Dentistry, İzmir, Turkey; 2 Private Practice, Istanbul, Turkey

## Abstract

**Objectives:**

The aim of this study was to compare the 2-year clinical performance of a bulk-fill composite resin and a nano-hybrid-filled composite resin in 6-12-year-old children in a split-mouth design.

**Materials and methods:**

This randomized, split-mouth, and double-blind study was conducted on 89 patients aged 6–12 years with caries on bilateral mandibular first molars. In a *split-mouth* design, restorations of mandibular permanent molars were completed with nano-hybrid organically modified ceramic (ORMOCER)-based bulk-fill composite resin Admira Fusion x-tra (Voco GmbH, Cuxhaven, Germany) and nano-hybrid composite Grandio (Voco, Cuxhaven, Germany). Futurabond U single dose (Voco, Cuxhaven, Germany) was used with selective enamel etching. The clinical success of the restorations was evaluated using USPHS and FDI criteria at 6, 12, and 24-month follow-up controls.

**Results:**

In the 2-year follow-up, all restorations were clinically acceptable. Grandio was significantly worse than Admira Fusion x-tra in terms of surface luster and superficial change (*p* < 0.05). Surface staining and color match scores increased in Admira Fusion x-tra compared with Grandio significantly (*p* < 0.05).

**Conclusions:**

Although both materials showed acceptable clinical performance over 2 years, a significant difference was observed between the surface luster, surface staining, marginal adaptation, and staining of the nano-hybrid composite placed with the incremental technique and the bulk-fill ORMOCER-based composite resin.

**Clinical relevance:**

As an alternative to nano-hybrid composite resins, using bulk-fill restorative materials, which can be indicated in the proper case, may contribute to shortening treatment procedures and increasing patient and physician comfort, leading to clinical success.

## Introduction

In dental clinical routine, composite resins are the most used esthetic restorative materials to restore permanent teeth in adult and pediatric patients. While the manufacturers are conducting various research in order to increase the clinical success of composite resins, they also consider user-friendly materials that are not sensitive to technique and have a short application time in clinical practice [1].

Composite resins can be classified based on the filler size. Nowadays, nano and micro-sized fillers which are glass (borosilicate), quartz (crystalline silica), colloidal silica, and pyrogenic silicon dioxide are used in the structure of most composite resins [2, 3]. Hybrid composite resins contain inorganic fillers of different sizes; the submicron-sized particulate (40 nm) is dispersed among the larger particles (10–50 μm). The distribution of different particle sizes is necessary to incorporate a maximum amount of filler into a resin matrix which consequently improves the mechanical properties of the composite resins [4]. Research that evaluated polymerization shrinkage focused on reducing particle sizes and changing the monomeric resin formulation based on nanotechnology to increase the filler amount [5]. Nano-hybrid and micro-hybrid composite resins contain pre-polymerized fillers, and nano and micro particles, respectively. It was reported that nano-hybrid and micro-hybrid composite resins were similar in terms of elastic strength and modulus and were clinically more successful th an micro-filled composites [6].

Another classification for composite resins is based on application technique. Bulk-fill composites are applied in a single layer of 4–5 mm, instead of the incremental technique. Bulk–fill composite resins differ in terms of application areas, viscosities, photoinitiator systems and monomer chemistries [7–9]. ORMOCER based bulk-fill composites have an organic structure that is changed into an inorganic-organic hybrid polymer forming a siloxane network. ORMOCER technology results in a three-dimensional polymerized structure with a reduced organic phase of composite resin compared to conventional composite resins. Therefore, it was reported that ORMOCER materials provide less polymerization shrinkage, better abrasion resistance, increased opacity, better polishability and biocompatibility [10–12]. Also, the absence of free monomers is one of the most important advantages of these materials.

In the incremental technique, composite resins are placed in the cavity with a maximum of 2 mm thickness layers. The rationale behind employing the incremental technique lies in achieving optimal polymerization throughout all sections of the composite material while simultaneously minimizing polymerization shrinkage for better physical properties, better edge matching, and reduction of cytotoxicity [13]. Bulk-fill composite resins have been formulated to facilitate a simplified application process in terms of both time efficiency and the attainment of technique-sensitive success, as they enable the application in a single layer with a thickness of 4–5 mm [14]. In bulk-fill composite resin materials, it is aimed to increase the depth of polymerization by using macro-size particles, increasing the translucency, and adding more sensitive photoinitiators [7, 15]. Besides, it is aimed to reduce polymerization shrinkage by adding modified monomers that modulate the setting reaction [16]. It has been reported that the lesser number of layers decreases the risk of gap occurrence and contamination between the layers, the difficulty in placing the material in conservative cavities, and chair time. Also, a more homogeneous structure can be achieved [16, 17]. Bulk-fill composite resins were reported to exhibit less polymerization shrinkage, cuspal deflection and microleakage than conventional resins [18, 19]. As a result of previous clinical studies, the 12–72 months clinical success of bulk-fill resin composites in the posterior region was similar to conventional resin composites placed with the incremental technique [1, 14, 20, 21].

Bulk-fill materials, which have a short clinical application period and are less sensitive to technique, are seen as an excellent alternative restorative material, especially in terms of ensuring and maintaining treatment compliance in children [1, 22]. This is the first study comparing ORMOCER-based bulk fill composite resins and nano-hybrid composite resins used for permanent teeth in a pediatric population. The null hypothesis of the study was “there is no significant difference between the 2-year clinical success of ceramic-containing bulk-fill composite resins and nano-hybrid composite resins in class I and II cavities of mandibular permanent molars in pediatric patients”.

## Materials and methods

This study was conducted according to the Helsinki Declaration and CONSORT standards and approved by İzmir Katip Çelebi University Faculty of Medicine Clinical Studies Local Ethics Committee. All patients and their legal guardians were informed about the clinical procedures, and they signed informed consent. The study was registered at ClinicalTrials.gov (NCT05432258).

### Study design, randomization and blinding

In a *split-mouth*, cross-controlled and double-blind design, 89 patients aged between 6 and 12 years were included. Using the splint-mouth design, the variation of factors associated with patients such as tooth localization in the arch, occlusal forces, and oral hygiene could be reduced. Their 2 mandibular first permanent molars were randomly divided into 2 groups based on materials and then 2 subgroups based on the sequence of the materials. To determine which material is applied to which side of the mandible and which site is treated first, randomization was done using software (the Research Randomizer® Version 4.0) by a pediatric dentist who was not one of the authors of this study. In each patient, one mandibular first permanent molar was restored using ORMOCER-based bulk-fill filling material Admira Fusion x-tra (Voco GmbH, Cuxhaven, Germany), and the contralateral was restored using nano-hybrid composite Grandio (Voco, Cuxhaven, Germany). A universal adhesive Futurabond U single dose (Voco, Cuxhaven, Germany) was used for all restorations, in the selective enamel etching mode. For the double-blind design, 2 different case report forms were filled in during the restoration session. The examiner and the patients were not informed about the restorative material used.

### Sample size calculation

The sample size was calculated using the NCSS/PASS program. Using data from a previous similar study, the initial “Alpha” score for marginal adaptation was assumed to be 100%, and the prevalence of individuals with Alpha for the 24th month was 86%. For the Mc Nemar Chi-square test, with 90% power, and a type 1 error level of 5%, the sample size was calculated as 74 [23]. Due to the 20% loss in follow-up in similar studies, it was aimed to reach 89 people by increasing the sample size at this rate.

### Inclusion criteria

Patients who have bilateral permanent first molars with Black class I or II primary caries that clinically scored 3, 4, or 5 according to the ICDAS II (International Caries Detection and Evaluation System); and radiographically D2-RB4 or D3-RC5 levels, scored 0 or 1 for the plaque index (Silnes and Löe, 1964), and 0 for gingival index (Löe and Silness, 1963) were included in this study.

### Exclusion criteria

Patients who were scored 1 and 2 on the Frankl behavioral scale; have any systemic disease that prevents the treatment (ASA 2, 3, 4, 5, 6); did not comply with their appointments; whose permanent first mandibular molars indicated root canal treatment, renewal of a restoration, or did not have antagonist teeth were excluded. Patients or their legal guardians who refused to participate in the study were also excluded.

### Clinic procedure

All restorations were completed under local anesthesia and rubber dam isolation. Caries were removed with an aerotor and micromotor. The cavity depth was assessed with a periodontal probe to determine the layer amount. In class II cavity, automatrix (SuperMat Adapt SuperCap Matrix, Kerr, Orange, ABD) and wooden wedges were used. 35% orthophosphoric acid gel (Vococid, Voco, Cuxhaven, Germany) was applied to the enamel surfaces for 20 s, and water rinsing for 20 s and air drying until the chalky-white enamel appearance were performed. Futurabond U single dose (Voco, Cuxhaven, Germany) universal adhesive was used according to the manufacturer’s instructions. The universal adhesive was activated and applied for 20 s and dried for 5 s, then polymerized for 10 s using light-emitting diode (VALO, Ultradent, USA) in standard power mode.

178 teeth in 89 patients were divided into 2 groups randomly. In each patient, 2 restorations were performed at 1-week intervals.

- Grandio (control group): 89 first mandibular molars restored with up to 2 mm oblique layers of nano-hybrid composite resin material (Grandio Shade A2, VoCo, Cuxhaven, Germany).
- Admira Fusion x-tra (study group): 89 first mandibular molar restored with up to 4 mm increments of nano-hybrid bulk-fill restorative material with ceramic filler (Admira Fusion x-tra, shade U, V56866; Voco GmbH, Cuxhaven, Germany) (Table 1).

Occlusal reduction, finishing and polishing were made using One Gloss Shofu Dental, Kyoto, Japan. Final polymerization was done using the VALO for 20 s in standard power mode.

**Table 1:** The composition and type of the materials used in this study

Materials	Manufacturer	Type	Composition
Admira fusion xtra (Batch no: 1914528)	VOCO, Cuxhaven, Germany	Bulkfill nano-hybrid ORMOCER	ORMOCER, photoinitiators, pigments, barium aluminum borosilicate glass, pyrogenic sílica (20–50 nm)
Grandio (Batch no: 1911375)	VOCO, Cuxhaven, Germany	Universal nano-hybrid restorative material	Methacrylate matrix (Bis-GMA, TEGDMA), inorganic filler (71.4 vol %)
Futurabond-U (Batch no: 1917243)	VOCO, Cuxhaven, Germany	Self-etch, selective-etch or total-etch	HEMA, Bis-GMA, HEDMA, MDP, UDMA, initiator, catalyst, and ethanol

The patients and their legal guardians were informed about the need for oral examinations and preventive interventions at the 6th, 12th, 18th and 24th months due to the high or moderate caries risk. They were motivated for follow-up sessions by being informed that new or secondary caries would be treated during the follow-up sessions if needed.

### Clinical performance evaluation

The clinical performance of the restorations will be evaluated with the criteria of the World Dental Federation (FDI) [24] and modified United States Public Health Service (USPHS) [25] at 6, 12, 18 and 24-month controls. A 10-year experienced pediatric dentist and a 4-year experienced dentist who was a postgraduate student in pediatric dentistry were calibrated about the USPHS and FDI evaluation criteria. Evaluations were made by these two calibrated clinicians who were unaware of the restorative materials used. Evaluating clinicians worked to be compatible with each other and were discussed until consensus if there was a controversy. Intraoral photographs were taken from the restored teeth at all control sessions.

In the evaluation according to FDI criteria, a suitable score was chosen from 5 different scores (clinically very good, clinically good (very good after editing), clinically adequate, clinically inadequate, and clinically poor) to determine esthetic, biological, and functional properties of the restorations (Table 2).

**Table 2:** FDI criteria used to evaluate the clinical performance of the restorations in this study

	A. Esthetic properties			B. Biological properties			C. Functional properties	
	Surface luster	Staining		Postoperative (hyper) sensitivity	Recurrence of caries (CAR), erosion, abfraction	Tooth integrity (enamel cracks, tooth fractures	Fracture of material and retention	Marginal adaptation
		Surface	Margin					
1. Clinically excellent / very good	Luster comparable to enamel.	No surface staining.	No marginal staining.	No hypersensitivity, normal vitality.	No secondary or primary caries.	Complete integrity.	No fractures / cracks.	Harmonious outline, no gaps, no white or discolored lines.
2. Clinically good (after polishing probably very good)	Slightly dull, not noticeable from speaking distance. Some isolated pores.	Minor surface staining, easily removable by polishing.	Minor marginal staining, easily removable by polishing.	Minor hypersensitivity for a limited period of time.	Small and localized demineralization.	Small marginal enamel fracture (< 150 μm). Hairline crack in enamel (< 150 μm).	Small hairline crack.	Marginal gap (< 150 μm). Small marginal fracture removable by polishing.
3. Clinically sufficient /satisfactory (minor shortcomings, no unacceptable effects but not adjustable w/o damage to the tooth)	Dull surface but acceptable if covered with film of saliva. Multiple pores on more than one third of the surface.	Moderate surface staining that may also present on other teeth, not esthetically unacceptable.	Moderate marginal staining, not esthetically unacceptable.	Moderate hypersensitivity Delayed/mild sensitivity; no subjective complaints, no treatment needed.	Larger areas of demineralization dentine not exposed. Only preventive measures necessary.	Marginal enamel defect (< 250 μm crack < 250 μm). Multiple cracks.	Two or more or larger hairline cracks and/or material chip fracture not affecting the marginal integrity.	Gap < 250 μm not removable. Several small marginal fractures.
4. Clinically unsatisfactory (but reparable)	Rough surface, cannot be masked by saliva film, simple polishing is not sufficient.	Unacceptable surface staining on the restoration and major intervention necessary for improvement.	Pronounced marginal staining; major intervention necessary for improvement.	Intense hypersensitivity. Delayed with minor subjective symptoms. No clinical detectable sensitivity. Intervention necessary but not replacement.	Caries with cavitation (localized and accessible and can be repaired).	Major marginal enamel defects; gap > 250 μm or dentine or base exposed. Large cracks > 250 μm, probe penetrates. Large enamel chipping or wall fracture.	Material chip fractures which damage marginal quality or approximal contacts. Bulk fractures with partial loss (less than half of the restoration).	Gap > 250 μm or dentine/base exposed. Severe ditching or marginal fractures. Larger irregularities or steps (repair necessary).
5. Clinically poor (replacement necessary)	Very rough, unacceptable plaque retentive surface.	Severe surface staining and/or subsurface staining, generalized or localized, not accessible for intervention.	Deep marginal staining, not accessible for intervention.	Intense, acute pulpitis or non vital tooth. Endodontic treatment is necessary, and restoration has to be replaced.	Deep caries or exposed dentine that is not accessible for repair of restoration.	Cusp or tooth fracture.	Partial or complete loss of restoration or multiple fractures.	Restoration (complete or partial) is loose but in situ.

Retention, color match, marginal discoloration, anatomical form, marginal adaptation, secondary caries, postoperative sensitivity and surface roughness from the USPHS criteria were assessed in this study (Table 3). In the modified USPHS system [25] “Alpha” is the best acceptable level; “Bravo” is the condition that is considered clinically successful with some deficiencies and deformations and does not require any intervention; The score of “Charlie” indicates clinically unsuccessful conditions requiring restoration or replacement [26].

**Table 3:** United State Public Health Service (USPHSR) criteria

	Explanation	Evaluation		Explanation	Evaluation		Explanation	Evaluation
**Retention**	No loss of restorative material	Alfa (A)	**Anatomic form**	Restoration is continuous with the existing anatomical form	Alfa (A)	**Postoperative hypersensitivity**	There is no sensitivity.	Alfa (A)
	Restoration is dropped or broken	Charlie (C)		Restoration is not the same as the anatomical form but acceptable amount	Bravo (B)		There is tender but temporary sensitivity.	Bravo (B)
**Color match**	There is no incompatibility	Alfa (A)		In the case where the anatomical form is insufficient with the dentin exposed	Charlie (C)		There is intolerable sensitivity	Charlie (C)
	There is marginal coloration, limited and not widespread	Bravo (B)	**Marginal adaptation**	The adaptation of the restoration to the tooth is very good. No stuttering when controlling with probe	Alfa (A)	**Superficial change**	It is smooth, similar to the enamel surface.	Alfa (A)
	Significant marginal coloration, penetrating into the pulp	Charlie (C)		In probe examination, the probe is inserted, but the dentin is not exposed	Bravo (B)		The surface is slightly rough.	Bravo (B)
**Cavosurface marginal discoloration**	No marginal coloration	Alfa (A)		The probe is inserted and moves into the recesses that extend to the enamel-dentin junction	Charlie (C)		The probe is rough enough to snap when swung around.	Charlie (C)
	Marginal coloration is present but limited, not extensive	Bravo (B)	**Secondary caries**	There is no softness and secondary caries at the marginal edge.	Alfa (A)			
	There is prominent, marginal discoloration reaching the pulp chamber	Charlie (C)		Secondary caries developed	Charlie (C)			

### Statistical analysis

Statistical analysis was performed with SPSS Statistics 25 (IBM, Armonk, New York). Categorical variables with their percentages and continuous variables with their mean and standard deviations were presented. Differences between baseline and follow-up percentages were evaluated with the McNemar Chi-Square and Cochran’s Q test. Percentage differences in the success of restorative materials at the same monitoring point were evaluated with the Chi-square test. Fischer’s exact test was used if there are less than 5 observations in one of the expected wells. Baseline parameters (ICDAS.II, DMFS/dmfs, radiographic score) were compared between material groups using the Mann Whitney U test, and Spearman Correlation Coefficient values were calculated. *p* < 0.05 will be considered significant.

## Results

### Study population

Of 102 pediatric dental patients, 89 who met the inclusion criteria were included in this study. In a split-mouth design, a total of 178 restorations were completed. 14 patients were lost to follow-up because of anxiety related to COVID-19 transmission (*n* = 9) and moving to another city (*n* = 5). The mean age was 9.61 (min = 6, max = 13) and the mean DMFS/dmfs was 9.43 (min = 3, max = 13).

The differences in ICDAS II score (mean diff.;0.027. *p* = 0.845), DMFS/dmfs (mean diff.;0.00. *p* = 1.000) and radiological score (mean diff.; 0.03. *p* = 0.703) were not statistically significant between material groups. The correlation values for each parameter between material groups were *r* = 0.961, *r* = 1.000 and *r* = 0.929; respectively.

### FDI criteria

Grandio showed a significant difference in terms of surface luster between T0 and T1, T2, T3, T4 (*p* < 0.001) while there was no significant difference in Admira Fusion x-tra through the time. In the comparison of the two materials, the surface luster of Grandio was significantly worse than Admira Fusion x-tra in T1, T2, T3, T4 (*p* < 0.001). For surface staining, there were significant differences between all periods in Grandio (*p* < 0.05) and Admira Fusion x-tra (*p* < 0.05). Surface staining of Admira Fusion x-tra significantly increased in T1 and T4 in comparison with Grandio (*p* < 0.05).

There was no difference regarding the recurrence of caries and tooth integrity in Grandio. However, in Admira Fusion x-tra, there were differences between baseline and T2 (*p* = 0.034), T3 (*p* = 0.023), T4 (*p* = 0.038) regarding the recurrence of caries and also between baseline and T3 (*p* = 0.011), T4 (*p* = 0.011), and also between T1 and T3 (*p* = 0.025), T4 (*p* = 0.025) regarding tooth integrity (Table 4).

**Table 4:** Clinical evaluation results of the restorations according to the FDI criteria

			Nano-hybrid composite resin (Grandio)					Bulk-fill composite resin (Admira Fusion x-tra)				
			T0	T1	T2	T3	T4	T0	T1	T2	T3	T4
FDI Criteria	Surface luster	1	75 (100)	7 (9.3)*^b^	3 (4)*^b^	3 (4)*^b^	4 (5.3)*^b^	75 (100)	71 (94.7)^a^	72 (96.0)^a^	72 (96.0)^a^	72 (96.0)^a^
		2	-	65 (86.7)*^a^	65 (86.7)*^a^	69 (92)*^a^	66 (88)*^a^	-	4 (5.3)^b^	3 (4.0)^b^	3 (4.0)^b^	3 (4.0)^b^
		3	-	3 (4)	3 (4)	3 (4)	5 (6.7)	-	-	-	-	-
		4	-	-	-	-	-	-	-	-	-	-
		5	-	-	-	-	-	-	-	-	-	-
	Surface staining	1	75 (100)	62 (82.7)*^b^	60 (80)*	57 (76)*	55 (73.3)*^b^	75 (100)	71 (94.7)*^a^	66 (88.0)*	66 (88.0)*	65 (86.7)*^a^
		2	-	13 (17.3)*^a^	15 (20)*	18 (24)*	20 (26.7)*^a^	-	4 (5.3)*^b^	9 (12.0)*	9 (12.0)*	10 (13.3)*^b^
		3	-	-	-	-	-	-	-	-	-	-
		4	-	-	-	-	-	-	-	-	-	-
		5	-	-	-	-	-	-	-	-	-	-
	Marginal staining	1	75 (100)	57 (76.0)*	48 (64.0)*	42 (56.0)*	41 (54.7)*	75 (100)	61 (81.3)*	53 (70.7)*	48 (64.0)*	48 (64.0)*
		2	-	17 (22.7)*	27 (36.0)*	33 (44.0)*	34 (45.3)*	-	14 (18.7)*	22 (29.3)*	27 (36.0)*	27 (36.0)*
		3	-	1 (1.3)*				-				
		4	-					-				
		5	-					-				
	Postoperative (hyper- sensitivity)	1	75 (100)	74 (98.7)	74 (98.7)	74 (98.7)	74 (98.7)	75 (100)	75 (100)	75 (100)	75 (100)	75 (100)
		2	-	1 (1.3)	-	-	-	-	-	-	-	-
		3	-		1 (1.3)	1 (1.3)	1 (1.3)	-	-	-	-	-
		4	-					-	-	-	-	-
		5	-					-	-	-	-	-
	Recurrence of caries (CAR), erosion, abfraction	1	75 (100)	73 (97.3)	72 (96)	72 (96)	72 (96)	75 (100)	72 (96.0)	70 (93.3)*	69 (92.0)*	69 (92.0)*
		2	-	2 (2.7)	3 (4)	3 (4)	3 (4)	-	2 (2.7)	4 (5.3)*	4 (5.3)*	4 (5.3)*
		3	-					-	-	-	-	-
		4	-					-	1 (1.3)	1 (1.3)*	2 (2.7)*	2 (2.7)*
		5	-					-				
	Tooth integrity (enamel cracks, tooth fractures)	1	74 (100)	74 (98.7)	74 (98.7)	73 (97.3)	73 (97.3)	75 (100)	73 (97.3)	71 (94.7)	68 (90.7)*	68 (90.7)*
		2	-	1 (1.3)	1 (1.3)	2 (2.7)	2 (2.7)	-	1 (1.3)	3 (4.0)	6 (8.0)*	6 (8.0)*
		3	-					-				
		4	-					-	1 (1.3)	1 (1.3)	1 (1.3)	1 (1.3)
		5	-					-				
	Fracture of material and retention	1	75 (100)	68 (90.7)	65 (86.7)	63 (84.0)	63 (84.0)	75 (100)	69 (92.0)*	66 (88.0)*	65 (86.7)*	65 (86.7)*
		2	-	7 (9.3)	10 (13.3)	12 (16.0)	12 (16.0)	-	6 (8.0)*	9 (12.0)*	8 (10.7)*	8 (10.7)*
		3	-					-			1 (1.3)*	1 (1.3)*
		4	-					-			1 (1.3)*	1 (1.3)*
		5	-					-				
	Marginal adaptation	1	75 (100)	71 (94.7)*	61 (81.3)*	44 (58.7)*	44 (58.7)*	75 (100)	70 (93.3)*	61 (81.3)*	40 (53.3)*	38 (50.7)*
		2	-	3 (4.0)*	13 (17.3)*	30 (40.0)*	30 (40.0)*	-	4 (5.3)*	13 (17.3)*	31 (41.3)*	33 (44.0)*
		3	-	1 (1.3)*	1 (1.3)*	1 (1.3)*	1 (1.3)*	-	-	-	3 (4.0)*	3 (4.0)*
		4	-					-	1 (1.3)	1 (1.3)	1 (1.3)*	1 (1.3)*
		5	-					-				

*statistically significant difference compared to T0

^a^statistically significant high in comparison of two materials in same follow-up

^b^statistically significant low in comparison of two materials in same follow-up.

Marginal staining and marginal adaptation increased over time in both groups. Marginal staining, postoperative hypersensitivity, recurrence of caries, tooth integrity, fracture of material and retention, and marginal adaptation were similar while comparing the two materials in the same period.

Postoperative hypersensitivity did not show a difference through the time periods between Grandio and Admira Fusion x-tra. In T1 (*p* = 0.014), T2 (*p* = 0.003), T3 (*p* = 0.003), and T4 (*p* = 0.005), fracture of material and retention were different from those assessed at baseline for Admira Fusion x-tra (Table 4).

### USPHS criteria

For Grandio, retention, secondary caries and postoperative hypersensitivity did not change over time. For Admira Fusion x-tra retention, post-op hypersensitivity and superficial change were not different between the time periods. Secondary caries were significantly different between T0 and T2, T3 in Admira Fusion x-tra. Superficial change scores of Grandio significantly increased in T1, T2, T3 and T4 in compared to Admira Fusion x-tra (Table 5).

**Table 5:** Clinical evaluation results of the restorations according to the USPHS criteria

			Nano-hybrid composite resin (Grandio)					Bulk-fill composite resin (Admira Fusion x-tra)				
			T0	T1	T2	T3	T4	T0	T1	T2	T3	T4
USPHS Criteria	Retention	A	75 (100)	74 (98.7)	74 (98.7)	74 (98.7)	74 (98.7)	75 (100)	75 (100)	75 (100)	74 (98.7)	74 (98.7)
		C	-	1 (1.3)	1 (1.3)	1 (1.3)	1 (1.3)	-	-	-	1 (1.3)	1 (1.3)
	Color match	A	75 (100)	69 (92.0)*	65 (86.7)*	62 (82.7)*	61 (81.3)*	75 (100)	74 (98.7)*	70 (93.3)*	70 (93.3)*	70 (93.3)*
		B	-	6 (8.0)*	10 (13.3)*	13 (17.3)*	14 (18.7)*	-	1 (1.3)*	5 (6.7)*	5 (6.7)*	5 (6.7)*
		C	-					-				
	Cavosurface marginal discoloration	A	75 (100)	58 (77.3)*	48 (64.0)*	42 (56.0)*	42 (56.0)*	75 (100)	61 (81.3)*	51 (68.0)*	45 (60.0)*	45 (60.0)*
		B	-	17 (22.7)*	27 (36.0)*	33 (44.0)*	33 (44.0)*	-	14 (18.7)*	24 (32.0)*	29 (38.7)*	29 (38.7)*
		C	-					-			1 (1.3)*	1 (1.3)*
	Anatomic form	A	75 (100)	70 (93.3)*	69 (92.0)*	69 (92.0)*	69 (92.0)*	75 (100)	71 (94.7)*	71 (94.7)*	67 (89.3)*	67 (89.3)*
		B	-	5 (6.7)*	6 (8.0)*	6 (8.0)*	6 (8.0)*	-	4 (5.3)*	4 (5.3)*	7 (9.3)*	7 (9.3)*
		C	-					-			1 (1.3)*	1 (1.3)*
	Marginal adaptation	A	75 (100)	55 (73.3)*	47 (62.7)*	34 (45.3)*	34 (45.3)*	75 (100)	61 (81.3)*	48 (64.0)*	29 (38.7)*	29 (38.7)*
		B	-	20 (26.7)*	28 (37.3)*	41 (54.7)*	41 (54.7)*	-	13 (17.3)*	26 (34.7)*	45 (60.0)*	45 (60.0)*
		C	-					-	1 (1.3)*	1 (1.3)*	1 (1.3)*	1 (1.3)*
	Secondary caries	A	75 (100)	73 (97.3)	72 (96.0)	72 (96.0)	72 (96.0)	75 (100)	73 (97.3)	71 (94.7)*	71 (94.7)*	71 (94.7)
		C	-	2 (2.7)	3 (4.0)	3 (4.0)	3 (4.0)	-	2 (2.7)	4 (5.3)	4 (5.3)	4 (5.3)
	Post operative hypersensitivity	A	75 (100)	74 (98.7)	74 (98.7)	74 (98.7)	73 (97.3)	75 (100)	75 (100)	75 (100)	75 (100)	75 (100)
		B	-	1 (1.3)	1 (1.3)	1 (1.3)	2 (2.7)	-	-	-	-	-
		C	-					-	-	-	-	-
	Superficial change	A	75 (100)	7 (9.3)*^b^	4 (5.3)*^b^	4 (5.3)*^b^	4 (5.3)*^b^	75 (100)	72 (96.0) ^a^	72 (96.0) ^a^	72 (96.0) ^a^	72 (96.0) ^a^
		B	-	68 (90.7)*^a^	71 (94.7)*^a^	71 (94.7)*^a^	71 (94.7)*^a^	-	3 (4.0)^b^	3 (4.0)^b^	3 (4.0) ^b^	3 (4.0) ^b^
		C	-					-				

*statistically significant difference compared to T0

^a^statistically significant high in comparison of two materials in same follow-up

^b^statistically significant low in comparison of two materials in same follow-up

There was a significant increase in the color match and cavosurface marginal discoloration and marginal adaptation of both materials over time. In the comparison of 2 materials in color match, Admira Fusion x-tra was significantly worse in T3 and T4. However, there was no difference in terms of cavosurface marginal discoloration and marginal adaptation between the two groups (Table 5).

For the Anatomic form of Grandio and Admira Fusion x-tra, there was a significant difference between T0 and T1 (0.046, 0.025), T2 (0.046, 0.014), T3 (0.007, 0.014). T4 (0.011, 0.014). The two materials were not different in terms of anatomic form at all time periods (Table 5).

## Discussion

In this study, the clinical performance of the ceramic-filled nanohybrid bulk-fill restorative material and a nano-hybrid composite resin which were used to restore class I and class II cavities of the bilateral first permanent molars of pediatric patients aged 6–12 years, differed in the 2-year follow-up. Surface luster, surface staining, color match, and superficial change were significantly different among the two materials, so the null hypothesis of this study was rejected.

Bulk-fill composite resins have advantages over interfacial gap, polymerization shrinkage and clinical practice time due to the decreased number of increments and chemical construction [27–29]. In pediatric dentistry practice, when making decisions among restoration materials for treating young permanent teeth, not only user-friendly properties but also the long-term performance of the materials should be considered. Therefore, bulk-fill restorative materials, which are better at chair time, polymerization shrinkage, cuspal movement, and less sensitive to technique; are revealed as an ideal alternative material for pediatric dental patients [4, 13, 14]. However, in the literature, a limited number of studies have evaluated bulk-fill composite resin in permanent teeth in pediatric patients.

In a 2-year follow-up of a previous study, the surface texture score of Grandio was Bravo of 26% [23]. Similarly, surface luster and superficial change scores were significantly increased in Grandio (nano-hybrid composite resin) compared to the Admira Fusion x-tra (ORMOCER-based bulk-filled composite resin) group in this study. This change can be explained by a Scanning Electron Microscope (SEM) evaluation which demonstrated surface deterioration through the disintegration of the organic matrix and exposure of the inorganic phase in the Grandio material [30]. While the Grandio showed a significant increase in surface roughness over time, there was no significant change in Admira Fusion x-tra in this study. Clinical success in the surface luster of ORMOCER-based bulk-fill composite might increase as a result of less organic phase and free monomers and better abrasion resistance of the material [10–12].

Although surface roughness was significantly higher in Grandio group compared to Admira Fusion x-tra, there was no difference in terms of secondary caries between the two groups. Conversely, the recurrence of caries increased in Admira Fusion x-tra group. This may be explained by the significant increase in fracture and tooth integrity scores in the same group over time. A previous study supporting our results stated that surface roughness was not associated with bacterial adhesion [31].

Staining of composite resins can be associated with the content of the materials, such as organic matrix, filler amount, and size [32, 33]. It was reported that an ORMOCER-based composite resin (Admira, Voco) showed more staining compared to a nano-hybrid composite resin (GrandioSO, Voco) [10]. While we used the bulk-fill form of the material in the present study, we observed similar results. Although surface staining scores were higher in Admira fusion x-tra compared to Grandio in 6 months and 24 months, a significant increase was observed in both materials over time. In a study compared Admira Fusion x-tra and methacrylate-based resin composite Omnichroma (2 one-shade materials), although the color stability was slightly worse in Admira Fusion x-tra group, both materials showed unacceptable color stability [34]. These results may be explained by a possible absence of the ideal integration between the siloxane group and polymerized microfiller around the resin in the ORMOCER-based resin structure [35]. Besides, resin-based materials showed unacceptable discoloration due to the consumption of staining drinks and as a limitation of this study, we did not record the dietary habits of the participants [10].

In a study comparing the 4-year clinical performance of the nanofiller-free hybrid composite resin Tetric Ceram (Ivoclar Vivadent, Schaan, Liechtenstein) and the nano-hybrid composite resin Grandio (Voco, Cuxhaven, Germany) using USPHS criteria, no significant difference was found between the two materials [36]. Similar to our results, they reported that scores in color match, surface roughness, marginal integrity, and tooth integrity (only in Admira Fusion x-tra) increased over time. In Grandio group, due to the shorter follow-up period, tooth integrity did not differ significantly over time; it may be considered as a limitation of this study.

Marginal adaptation, marginal staining, and surface staining increased over time in both materials. Marginal adaptation of a nanohybrid composite resin (Grandio) showed better clinical performance compared to low shrinkage posterior composite (Quixfil, Denstply, Kostanz, Germany) in a study with a 2-year follow-up [23]. It was reported that ORMOCER-based bulk-fill composite resin (Admira Fusion x-tra) was similar to the ORMOCER-based composite resin (Admira) and better than other bulk-fill restorative materials based on marginal quality and microleakage in-vitro [18, 37]. In an in-vitro study, bulk-fill composite resins consisting of Admira Fusion x-tra showed lower polymerization shrinkage compared to the low-viscosity bulk-fill composite resins and conventional composite resins [27]. Consistent with the previous studies, Grandio and Admira Fusion x-tra showed clinically acceptable and similar marginal adaptation in this study.

In a 4-year study, decreased marginal adaptation was observed over time in all bulk-fill composite resin materials completed with incremental (2 mm) and bulk-fill (4 mm) techniques. Marginal adaptation and discoloration were not associated with the technique [38]. Contrary to these results, in a 36-month study, a bulk-fill composite resin showed better clinical performance than a nano-filled composite resin in terms of marginal adaptation and marginal discoloration [20]. This conflict might be associated with the difference in the composition of the materials. In the present study, marginal adaptation was not acceptable in one of the ORMOCER-based bulk-fill composite resin restorations. However, there was no significant difference between the two materials in terms of marginal adaptation for the USPHS and FDI criteria.

Postoperative sensitivity was recorded in only one participant and therefore, there was no significant difference between materials or sessions. In a recently published review and meta-analysis, no significant difference was reported between bulk-fill and incremental composite resin in postoperative hypersensitivity [39].

FDI criteria are well described and offer a wide range of scores for a more sensitive clinical evaluation. In previous studies, FDI criteria were reported to provide more sensitive determinations in short time periods due to the inclusion of 5 different scores [40, 41]. Although the FDI criteria were found to be more sensitive and reliable for clinical evaluation, no significant difference was found between the results of the FDI and USPHS criteria in this study.

So many *in-vitro* and clinical studies have been conducted using universal adhesives previously. According to the results of laboratory studies, it has been recommended that universal adhesives should be applied with selective enamel etching in permanent teeth and with a total-etch strategy in deciduous teeth [42]. Although it was reported that the etching mode (self-etch or total-etch) did not affect the clinical performance of the bonding agent in some clinical trials; for some bonding agents, marginal discoloration and marginal integrity were worse in the self-etch mode compared to total-etch mode [43–45]. Previous studies reported that selective enamel etching prevented marginal discoloration and enhanced marginal adaptation [40, 44]. Therefore, we preferred using the selective enamel etching mode of the same universal adhesive in all restorations to avoid the potential effects of different adhesive systems.

## Conclusion

Although both materials exhibited acceptable clinical performance over the 2-year period, the bulk-fill ORMOCER-based composite resin demonstrated superiority in terms of surface luster and superficial changes. However, it was worse in surface staining and color match compared to the nano-hybrid composite placed using the incremental technique. It should be taken into consideration that different results may arise with various composite resins having distinct compositions, especially when considering longer follow-up periods.

## Data Availability

Not applicable.

## References

[1] Durão M, de Andrade A, do Prado AKM (2021). Thirty-six-month clinical evaluation of posterior high-viscosity bulk-fill resin composite restorations in a high caries incidence population: interim results of a randomized clinical trial. Clin Oral Investig.

[2] Dayangaç B (2015) Kompozit Rezin Restorasyonlar. Quintessence, Ankara

[3] Lien W, Vandewalle KS (2010). Physical properties of a new silorane-based restorative system. Dent Mater: Off Publ Acad Dent Mater.

[4] Blackham JT, Vandewalle KS, Lien W (2009). Properties of hybrid resin composite systems containing prepolymerized filler particles. Oper Dent.

[5] Tomaszewska IM, Kearns JO, Ilie N, Fleming GJP (2015) Bulk fill restoratives: To cap or not to cap – That is the question? J Dent [Internet]. [cited 2019 May 18];43(3):309–16. Available from: http://www.ncbi.nlm.nih.gov/pubmed/25625673 10.1016/j.jdent.2015.01.010 25625673

[6] Ferracane JL (2011). Resin composite-state of the art. Dent Mater: Off Publ Acad Dent Mater.

[7] Ilie N, Stark K (2015). Effect of different curing protocols on the mechanical properties of low-viscosity bulk-fill composites. Clin Oral Investig.

[8] Czasch P, Ilie N (2013). In vitro comparison of mechanical properties and degree of cure of bulk fill composites. Clin Oral Investig.

[9] Walter R (2013). Bulk-fill flowable composite resins. J Esthet Restor Dent.

[10] Llena C, Fernández S, Forner L (2017). Color stability of nanohybrid resin-based composites, ormocers and compomers. Clin Oral Invest.

[11] Manhart J, Kunzelmann KH, Chen HY, Hickel R (2000). Mechanical properties of new composite restorative materials. J Biomed Mater Res.

[12] Yap AUJ, Lim LY, Yang TY (2005). Influence of dietary solvents on strength of nanofill and ormocer composites. Oper Dent.

[13] Poskus L, Placido E, Cardoso PEC (2004). Influence of placement techniques on Vickers and Knoop hardness of class II composite resin restorations. Dent Mater.

[14] Veloso SRM, Lemos CAA, de Moraes SLD (2019). Clinical performance of bulk-fill and conventional resin composite restorations in posterior teeth: a systematic review and meta-analysis. Clin Oral Investig.

[15] Benetti A, Havndrup-Pedersen C, Honoré D (2015). Bulk-fill resin composites: polymerization contraction, depth of cure, and gap formation. Oper Dent.

[16] Fronza BM, Rueggeberg FA, Braga RR (2015). Monomer conversion, microhardness, internal marginal adaptation, and shrinkage stress of bulk-fill resin composites. Dent Mater.

[17] El-Safty S, Akhtar R, Silikas N, Watts DC (2012). Nanomechanical properties of dental resin-composites. Dent Mater.

[18] Politi I, McHugh LEJ, Al-Fodeh RS, Fleming GJP (2018). Modification of the restoration protocol for resin-based composite (RBC) restoratives (conventional and bulk fill) on cuspal movement and microleakage score in molar teeth. Dent Mater.

[19] McHugh LEJ, Politi I, Al-Fodeh RS, Fleming GJP (2017). Implications of resin-based composite (RBC) restoration on cuspal deflection and microleakage score in molar teeth: Placement protocol and restorative material. Dent Mater.

[20] Yazici AR, Antonson SA, Kutuk ZB, Ergin E (2017). Thirty-six-month clinical comparison of bulk fill and nanofill composite restorations. Oper Dent.

[21] Yazici AR, Kutuk ZB, Ergin E (2022). Six-year clinical evaluation of bulk-fill and nanofill resin composite restorations. Clin Oral Investig.

[22] Atabek D, Aktaş N, Sakaryali D, Bani M (2017). Two-year clinical performance of sonic-resin placement system in posterior restorations. Quintessence Int (Berlin Germany: 1985).

[23] Arhun N, Celik C, Yamanel K (2010). Clinical evaluation of resin-based composites in posterior restorations: two-year results. Oper Dent.

[24] Hickel R, Peschke A, Tyas M (2010). FDI world dental federation: clinical criteria for the evaluation of direct and indirect restorations—update and clinical examples. Clin Oral Investig.

[25] Cvar JF, Ryge G (2005). Reprint of criteria for the clinical evaluation of dental restorative materials. Clin Oral Investig.

[26] Türkün LS, Türkün M, Ozata F (2003). Two-year clinical evaluation of a packable resin-based composite. J Am Dent Assoc.

[27] Rizzante FAP, Duque JA, Duarte MAH (2019). Polymerization shrinkage, microhardness and depth of cure of bulk fill resin composites. Dent Mater J.

[28] Kim RJ-Y, Kim Y-J, Choi N-S, Lee I-B (2015). Polymerization shrinkage, modulus, and shrinkage stress related to tooth-restoration interfacial debonding in bulk-fill composites. J Dent.

[29] Comba A, Baldi A, Michelotto Tempesta R (2022). Influence of curing Mode and Layering technique on the 3D interfacial gap of bulk-fill Resin composites in Deep Class-I restorations: a Micro-CT volumetric study. J Adhes Dent.

[30] Heintze SD, Forjanic M, Ohmiti K, Rousson V (2010). Surface deterioration of dental materials after simulated toothbrushing in relation to brushing time and load. Dent Mater.

[31] Bilgili D, Dündar A, Barutçugil Ç (2020). Surface properties and bacterial adhesion of bulk-fill composite resins. J Dent.

[32] Alawjali SS, Lui JL (2013). Effect of one-step polishing system on the color stability of nanocomposites. J Dent.

[33] Çakır Kılınç NN, Demirbuğa S (2023). The influence of different placement techniques on the clinical success of bulk-fill resin composites placed in class II cavities: a 4-year randomized controlled clinical study. Clin Oral Invest.

[34] Ebaya MM, Ali AI, El-Haliem HA, Mahmoud SH (2022). Color stability and surface roughness of ormocer- versus methacrylate-based single shade composite in anterior restoration. BMC Oral Health.

[35] Gregor L, Krejci I, Di Bella E, Feilzer AJ, Ardu S (2015) Silorane, ormocer, methacrylate and compomer long-term staining susceptibility using DE and DE00 colour-difference formulas. Odontology 2015. 10.1007/s10266-015-0212-7 10.1007/s10266-015-0212-7 26178651

[36] Krämer N, Reinelt C, Richter G (2009). Nanohybrid vs. fine hybrid composite in Class II cavities: clinical results and margin analysis after four years. Dent Mater.

[37] Signore A, Solimei L, Arakelyan MG (2021). Marginal quality of a full-body bulk-fill composite placed with an universal adhesive system in etch-and-rinse and self-etch mode: an in vitrostudy. J Clin Exp Dent.

[38] Çakır Kılınç NN, Demirbuğa S (2022). The influence of different placement techniques on the clinical success of bulk-fill resin composites placed in class II cavities: a 4-year randomized controlled clinical study. Clin Oral Investig.

[39] Kunz PVM, Wambier LM, Kaizer Md (2022). Is the clinical performance of composite resin restorations in posterior teeth similar if restored with incremental or bulk-filling techniques? A systematic review and meta-analysis. Clin Oral Invest.

[40] Perdigão J, Kose C, Mena-Serrano AP (2014). A new universal simplified adhesive: 18-month clinical evaluation. Oper Dent.

[41] De Paula E, Tay LY, Kose C, Mena-Serrano A (2015). Randomized clinical trial of four adhesion strategies in cervical lesions: 12-month results. Int J Esthet Dent.

[42] Nagarkar S, Theis-Mahon N, Perdigão J (2019). Universal dental adhesives: current status, laboratory testing, and clinical performance. J Biomed Mater Res B Appl Biomater.

[43] Çakır NN, Demirbuga S (2018) The effect of five different universal adhesives on the clinical success of class I restorations: 24-month clinical follow-up. Clin Oral Investig. 10.1007/s00784-018-2708-3 10.1007/s00784-018-2708-3 30368662

[44] Peumans M, De Munck J, Van Landuyt KL (2010). Eight-year clinical evaluation of a 2-step self-etch adhesive with and without selective enamel etching. Dent Mater.

[45] Can Say E, Özel E, Yurdagüven H, Soyman M (2014). Three-year clinical evaluation of a two-step self-etch adhesive with or without selective enamel etching in non-carious cervical sclerotic lesions. Clin Oral Invest.

